# Multi-resolution transfer learning for tampered image classification using SE-enhanced fused-MBConv and optimized CNN heads

**DOI:** 10.1038/s41598-025-17799-0

**Published:** 2025-09-24

**Authors:** Jithin Reddy Korsipati, Rama Muni Reddy Yanamala, Archana Pallakonda, Rayappa David Amar Raj, K. Krishna Prakasha

**Affiliations:** 1 Amrita School of Artificial Intelligence, Amrita Vishwa Vidyapeetham, Coimbatore, Tamil Nadu 641112 India; 2 Department of Electronics and Communication Engineering, Indian Institute of Information Technology Design and Manufacturing (IIITD&M) Kancheepuram, Chennai, 600127 India; 3 Department of Computer Science and Engineering, National Institute of Technology, Warangal, 506004 India; 4 Amrita School of Artificial Intelligence, Amrita Vishwa Vidyapeetham, Coimbatore, Tamil Nadu 641112 India; 5 Manipal Institute of Technology, Manipal Academy of Higher Education, Manipal, India

**Keywords:** Image forgery detection, EfficientNetV2, Fused-MBConv block, Squeeze-and-Excitation (SE) Attention, Focal loss optimization, Transfer learning, Engineering, Electrical and electronic engineering

## Abstract

The widespread use of digital image tampering has created a strong need for accurate and generalizable detection systems, especially in domains like forensics, journalism, and cybersecurity. Traditional handcrafted methods often fail to capture subtle manipulation artifacts, and many deep learning approaches lack generalization across diverse image sources and manipulation techniques. To address these limitations, we propose a tampered image classification model based on transfer learning using EfficientNetV2B0. This backbone is combined with a lightweight, regularized CNN classification head and optimized using Focal Loss to address class imbalance. The architecture integrates compound scaling, fused MBConv layers, and squeeze-and-excitation (SE) attention to improve feature representation and robustness. We evaluate the model on four benchmark datasets-CASIA v1, Columbia, MICC-F2000, and Defacto (Splicing)-and achieve exceptional performance, with AUC scores up to 1.0000 and F1-scores up to 0.9997. Comparisons with 42 state-of-the-art models, including IML-ViT, MVSS-Net++, ConvNeXtFF, and DRRU-Net, show our method consistently outperforms existing approaches in accuracy, precision, recall, and generalization, particularly on high-resolution and compressed images. These results demonstrate the practical effectiveness and forensic reliability of the proposed system.

## Introduction

Recognition of forged images is a critical task in fields such as forensics and cybersecurity. Conventional techniques often struggle to accurately identify tampered images due to complex artifacts introduced during tampering^1^. This paper introduces a deep learning model based on EfficientNetV2B0, a highly efficient convolutional neural network (CNN) architecture that achieves state-of-the-art performance in image classification tasks. By leveraging transfer learning, a regularized deep classification head, and Focal Loss, the model enhances classification performance and effectively handles class imbalance in tampered image datasets, building upon recent advances in multi-scale detection approaches^2^.

Image tampering detection and localization are vital in computer forensics due to the widespread manipulation of visual information in domains like social media, journalism, and law. Forgery techniques, including splicing, copy-move, and removal, can alter image semantics without leaving visible traces, posing significant threats to information authenticity. Traditional handcrafted methods rely on statistical differences, while modern deep learning models exploit spatial and contextual information for accurate detection. Localization further enhances forensic value by pinpointing manipulated areas. Despite progress, challenges remain in achieving real-time detection, robustness against post-processing, and generalization across diverse manipulation types.

Early image forgery detection research primarily utilized handcrafted features and statistical methods. For instance, the approach in^3^ combined Local Binary Patterns (LBP) with Discrete Cosine Transform (DCT) applied to chrominance channels to identify forgeries, achieving high accuracy on specific datasets but lacking localization and computational efficiency. Similarly,^4^ integrated Gabor wavelets and Local Phase Quantization (LPQ) with Non-negative Matrix Factorization (NMF), demonstrating strong rotation and scale invariance but limited by handcrafted texture descriptors, reducing adaptability to unseen forgeries. The method in^5^ employed GLCM-based statistical features with BDCT, performing well under compression and noise but failing to localize tampered areas. Other works, such as^6,7^, also relied on statistical descriptors like OELTP and DCT coefficient analysis, respectively, but were constrained by their lack of deep semantic understanding and localization capabilities.

Hybrid solutions emerged by combining classical preprocessing with learning-based classifiers. The method in^8^ proposed a CNN-DWT fusion system for enhanced splicing detection through feature complementarity, but it lacked robustness against adversarial attacks and localization. Similarly, Ref.^9^ fused CLAHE-boosted CNN features with SVM classification, achieving high accuracy but struggling with cross-dataset generalization and adversarial resistance. The approach in^10^ utilized Ripplet Transform for iris texture classification, offering effective directional detail but missing deep learning potential and spoofing resistance. The shift to deep learning-based models marked significant progress in forgery localization. The work in^11^ employed Vision Transformers (ViT) with the Segment Anything Model (SAM) for dual classification and localization, though computational overhead limited real-time applicability. The method in^12^ used a ResNet-50 backbone with multi-scale loss and CBAM attention for pixel-level splicing detection, improving boundary attention but faltering under low-quality compression. The ME-Net in^13^ enhanced edge localization with PSDA and EEPA modules in RGB and noise spaces, achieving state-of-the-art F1 scores but introducing complexity. Similarly, Ref.^14^ introduced a Multi-task FCN model with edge-enhanced inference for fine-grained localization, showing strong generalization but limited by high computational costs and reliance on pixel-level annotations.

Recent architectures increasingly incorporate attention mechanisms, pyramid-based fusion, and multi-branch approaches. The method in^15^ used multi-scale ConvNeXt backbones with feature pyramid fusion and loss adaptation for improved tamper boundary detection, though fixed input resolution and high resource demands posed deployment challenges. The LBRT model in^16^ introduced a transformer-based dual-branch approach with global-local attention and intra-patch refinement for copy-move detection, but it was sensitive to patch size and less robust against subtle manipulations. The CSR-Net in^17^ pioneered spline-based regression for boundary-aware localization, yet it was sensitive to thresholds and curve-fitting. The MAC-Net in^18^ employed dual attention modules and a new splicing dataset (SMI20K) for robust real-time localization, but it was limited to splicing cases without a lightweight extension.

Advanced models, such as those in^19–21^, significantly improve localization and feature fusion. The approach in^19^ combined boundary incoherence enhancement with hybrid ResNet-ViT modules, excelling in splicing, copy-move, and removal detection but at the cost of high complexity and latency. The method in^20^ utilized dual streams (MEA and MCS) for edge- and context-driven detection, achieving improved F1 scores but requiring heavy preprocessing, making it impractical for mobile forensics. The FARA-Net in^21^ integrated CNN and Transformer features with adaptive aggregation and region-aware learning, performing exceptionally across datasets but hindered by its heavy architecture for real-time use. Finally, the ESRNet in^22^ offered an end-to-end framework for splicing, copy-move, and removal detection with EFPN, RFBA, and GRA modules, achieving high processing speed (approximately 53 FPS) and robustness to compression and adversarial attacks, though challenges remained with synthetic training data and high-noise edge localization.

The Table 1 highlights the most critical limitations of various existing image forgery detection methods and demonstrates the superiority of the proposed model in addressing these limitations. Existing methods, conventionally depending on hand-designed features, statistical models, or hybrid models, are plagued by limitations including poor localization, inability to generalize to unseen forgery cases, and high computational cost. The proposed model, built on EfficientNetV2B0, SE-attention, and Focal Loss, presents a stronger, more generalizable, and computationally more efficient approach to image forgery detection for large manipulation categories and datasets. The table shows how the proposed model outperforms traditional methods in various important aspects including adversarial robustness, real-time capability, and ability to detect subtle manipulations.

**Table 1:** Comparison of existing approaches and proposed model for image forgery detection.

Drawback addressed	Existing approaches	Proposed model
Localization	Approaches like LBP + DCT (e.g.,^3^) achieve high accuracy but lack localization and computational efficiency	EfficientNetV2B0 backbone with SE-attention and Fused MBConv enhances feature extraction for better localization
Adaptability to unseen forgeries	Gabor wavelets, LPQ with NMF (e.g.,^4^) show strong rotation and scale invariance but are limited by handcrafted texture descriptors	Deep learning-based approach with transfer learning and Focal Loss, improving adaptability to unseen forgeries
Deep semantic understanding	Statistical methods like GLCM and BDCT (e.g.,^5^) perform well under noise but lack deep semantic understanding	The model integrates deep learning features, enhancing semantic understanding for forgery detection
Adversarial robustness	Hybrid systems like CNN-DWT (e.g.,^8^) lack robustness against adversarial attacks and localization	The proposed model is robust to adversarial attacks, enhancing generalization and localization across datasets
Real-time applicability	Vision Transformer (ViT) with SAM (e.g.,^11^) faces high computational overhead, limiting real-time applicability	The proposed model is optimized for real-time detection with minimal latency using lightweight architecture
Robustness under compression	ResNet-50 with multi-scale loss (e.g.,^12^) improves boundary attention but struggles under compression	EfficientNetV2B0 backbone with SE-attention and Focal Loss ensures robustness under compression and diverse manipulations
Generalization across datasets	Methods like CLAHE-boosted CNN + SVM (e.g.,^9^) struggle with cross-dataset generalization	The model outperforms 42 state-of-the-art methods, showing high generalization across diverse datasets and manipulation types
Complexity and latency	Complex models like ResNet-ViT hybrid (e.g.,^19^) are computationally expensive with high latency	The proposed model reduces complexity with a lightweight architecture while achieving high performance for real-time use
Handling subtle manipulations	MAC-Net (e.g.,^18^) is limited to splicing cases and struggles with subtle manipulations	The proposed model excels in detecting subtle manipulations using SE-attention and multi-resolution feature extraction
Rotation and scale invariance	Gabor wavelets + LPQ (e.g.,^4^) provide rotation and scale invariance but are limited by handcrafted features	The proposed model’s deep learning approach allows better feature extraction, improving performance on rotated and scaled images
Boundary awareness	ME-Net (e.g.,^13^) enhances edge localization but introduces complexity	EfficientNetV2B0 with SE-attention blocks improves boundary detection without additional complexity
Cross-resolution generalization	Models like ConvNeXtFF (e.g.,^15^) struggle with fixed input resolution and high resource demands	The proposed model generalizes across multiple image resolutions and manipulation types effectively
Handling noisy data	CSR-Net (e.g.,^17^) introduces spline-based regression but is sensitive to thresholds and curve-fitting	The model handles noisy data effectively with the integration of SE-attention and Focal Loss for robust learning
Generalization to various manipulation types	LBRT (e.g.,^16^) is limited to copy-move detection and struggles with subtle manipulations	The proposed model handles multiple forgery types (splicing, copy-move, hybrid) and performs well under varying conditions

The main contributions of proposed work are:

- A lightweight yet efficient tampering detection framework that integrates EfficientNetV2B0 as the backbone with a compact CNN classification head, leveraging compound scaling, fused MBConv, Squeeze-and-Excitation attention, and Focal Loss to address class imbalance and enhance feature representation.
- Comprehensive evaluation on four diverse benchmark datasets (CASIA v1, Columbia, MICC-F2000, and Defacto) covering multiple forgery types (splicing, copy-move, hybrid) and varying resolutions, ensuring robustness across real-world scenarios.
- Extensive performance assessment using accuracy, precision, recall, F1-score, and AUC, complemented by confusion matrices and ROC curves for detailed class-wise and threshold-based analysis.
- Comparative analysis against over 40 state-of-the-art methods (e.g., IML-ViT, ConvNeXtFF, DRRU-Net, MVSS-Net++), demonstrating consistent improvements in F1-score, AUC, and accuracy across all datasets.
- Both quantitative (metrics, ROC, confusion matrices) and qualitative (visual classification results) validations confirming reliability, with near-perfect detection performance on MICC-F2000 and Defacto, even under compression and diverse manipulation types.

## Dataset description

**Fig. 1:**
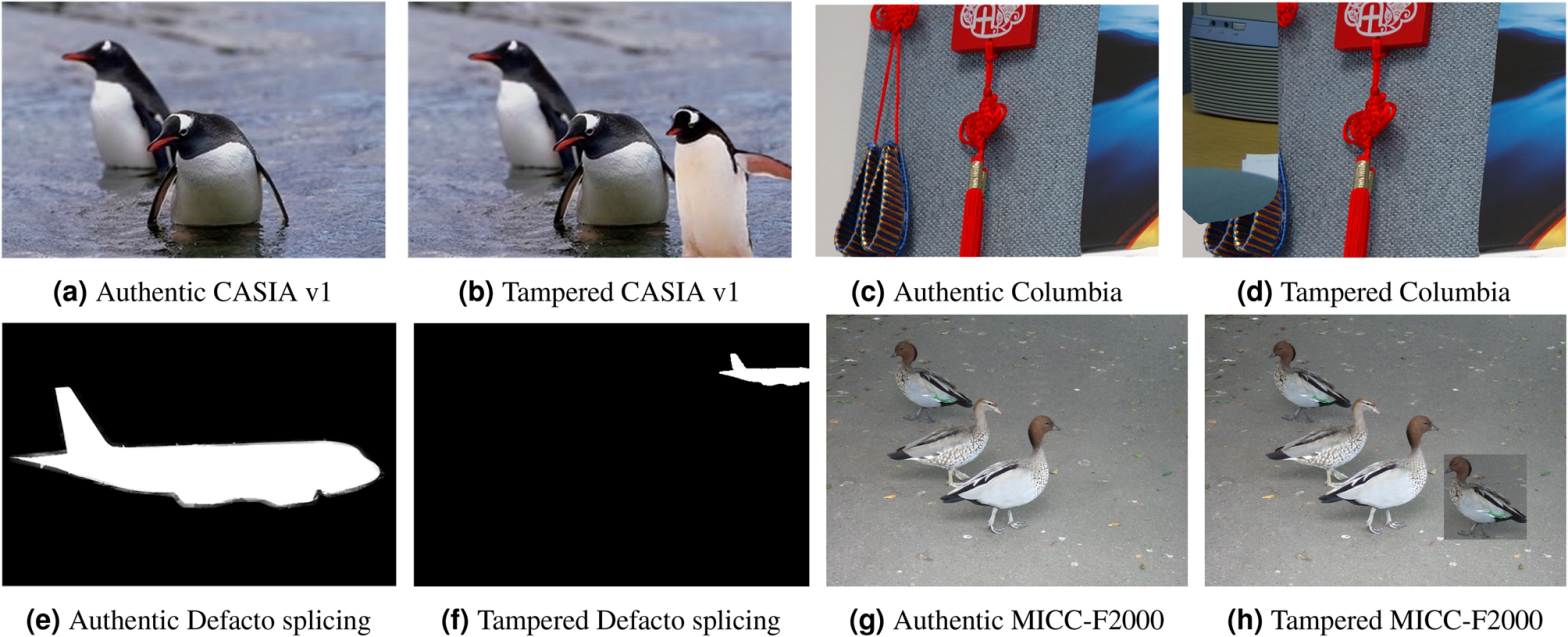
Examples of authentic and tampered images from the CASIA, Columbia, Defacto, and MICC-F2000 datasets.

In the domain of image forgery, datasets are critical for evaluating model performance and generalization. Widely used datasets include CASIA v1^23^, MICC-F2000^24^, Defacto (Splicing)^25^, and Columbia^26^, as shown in Table 2 each providing a mix of genuine and tampered images with ground truth masks that precisely demarcate manipulated regions. CASIA v1 contains 1721 JPEG images, comprising 800 authentic as shown in Fig. 1a and 921 tampered samples as shown in Fig. 1b , typically sized around 384$\times$256 pixels, in both grayscale and color, with splicing as the primary forgery type. Defacto (Splicing) includes 984 PNG images, evenly split between 492 authentic as shown in Fig. 1e and 492 tampered samples as shown in Fig. 1f , all at 512 $\times$ 512 resolution, focusing on splicing manipulations, making it suitable for evaluating models on this specific forgery type. MICC-F2000 consists of 300 high-resolution JPEG images, with 130 authentic as shown in Fig. 1g and 170 forged samples as shown in Fig. 1h , resolutions up to 2048 $\times$ 1536, and various manipulation types, accompanied by ground truth masks for localization. Columbia provides 363 TIFF images, with 183 authentic as shown in Fig. 1c and 180 forged samples as shown in Fig. 1d , all sized at 757 $\times$ 568, ideal for pixel-level detail analysis due to their uncompressed nature.

**Table 2:** Summaries of the datasets.

Data	Total	Authentic	Tampered	Format	Resolution	Manipulation
CASIA v1	1721	800	921	JPEG	384 $\times$ 256	Splicing
Defacto	984	492	492	PNG	512 $\times$ 512	Splicing
MICC-F2000	300	130	170	JPEG	2048 $\times$ 1536	Various
Columbia	363	183	180	TIFF	757 $\times$ 568	Splicing

For training, all images were resized uniformly to $224\times 224\times 3$ and normalized for consistency across the dataset. A training-validation split of 80 : 20 was used. Transfer learning was applied in the model architecture, initializing EfficientNetV2B0 with ImageNet-pretrained weights. The backbone layers were frozen during the early epochs to preserve general visual patterns, followed by fine-tuning of deeper layers to acquire patterns of forgery. Optimization was performed using the Adam optimizer with an initial learning rate of $1\times 10^{-3}$, dynamically reduced using a ReduceLROnPlateau scheduler based on AUC validation. Focal Loss with $\alpha = 0.25$ and $\gamma = 2.0$ was used to address class imbalance. Dropout with a rate of 0.5 and L2 weight decay were used as regularization methods in the classification head to avoid overfitting. Training was performed using PyTorch 1.13 with an average training time of 10–20 minutes per dataset for 50 epochs. The models were evaluated using performance metrics including accuracy, precision, recall, F1-score, AUC, confusion matrices, and ROC curves to measure performance comprehensively. To further enhance robustness and reduce overfitting, an on-the-fly data augmentation pipeline^27^, which primarily focuses on augmentation strategies, was applied to training images via a custom TensorFlow image data generator. The augmentation operations included horizontal flipping, random mirroring, small-angle random rotations ($\pm 10^\circ$), random translations along both axes, and brightness adjustment with clipping to prevent pixel overflow. Each transformation was applied with controlled probability to maintain diversity while preserving the structural and statistical integrity of tampered areas, ensuring that manipulation artifacts such as boundary inconsistency and blending traces remained identifiable. Additionally, class imbalance in certain datasets was handled by applying the RandomOverSampler method from the imbalanced-learn library, creating balanced batches of real and manipulated samples. The combined use of balancing and augmentation strategies improved the model’s ability to learn manipulation-agnostic features and to generalize across various datasets and manipulation types.

## Proposed model architecture

These datasets enable two primary evaluation types: image-level classification and pixel-level localization. Image-level classification determines whether an image is authentic or tampered, using metrics such as accuracy, precision, recall, F1-score, and AUC. Pixel-level localization identifies tampered regions within an image using ground truth masks, evaluated with metrics like Intersection over Union (IoU), pixel-wise F1-score, and mean Average Precision (mAP). To assess detection models’ robustness and adaptability, cross-dataset evaluation is recommended, ensuring models do not overfit to specific dataset characteristics and can generalize across various forgery scenarios and imaging conditions.

**Fig. 2:**
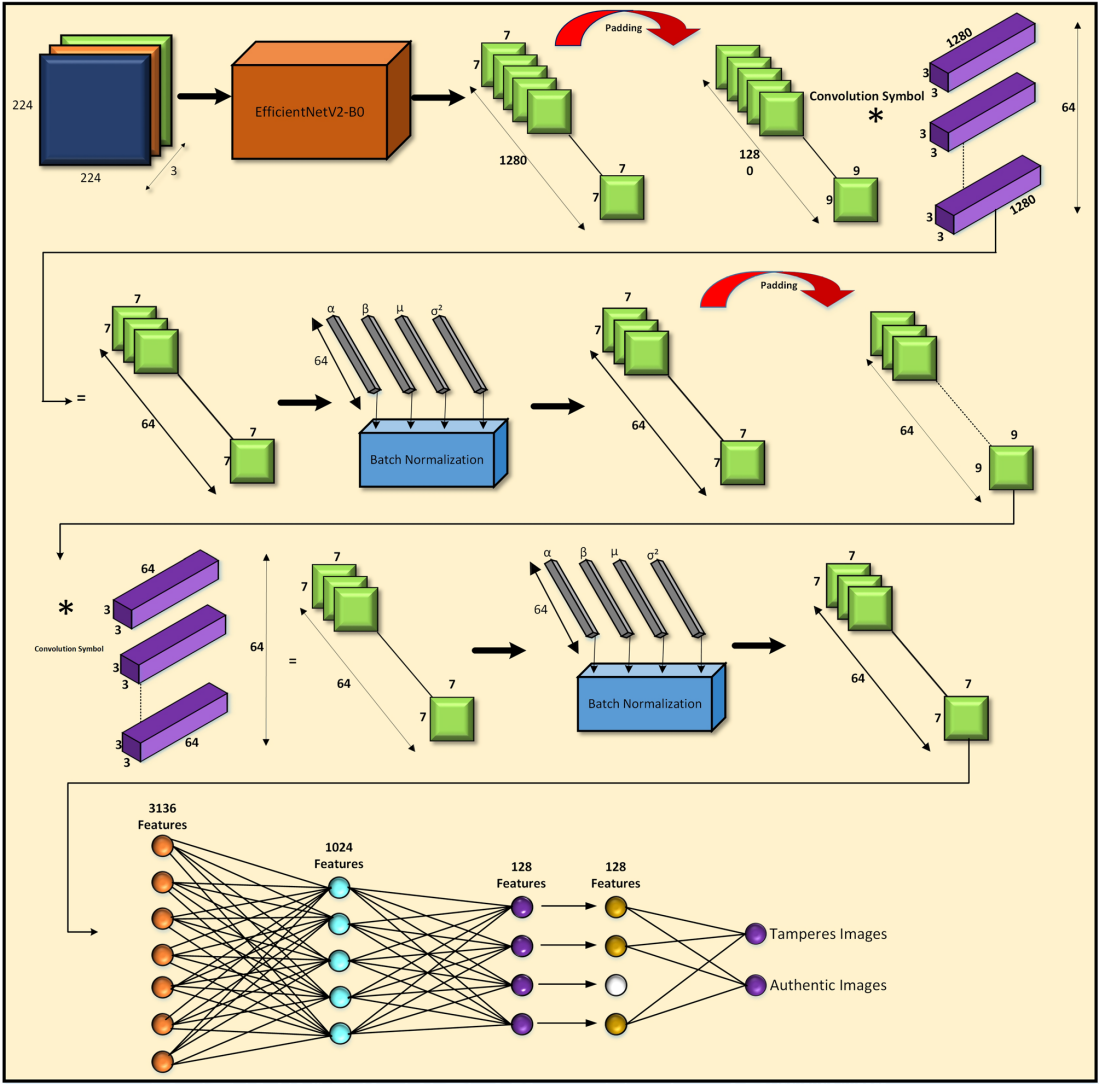
Overview architecture of proposed model.

The overview of the proposed model architecture as shown in Fig. 2 integrates a robust feature extractor, EfficientNetV2-B0, with a custom-designed convolutional and fully connected classification pipeline to distinguish between authentic and tampered images. The input image, sized at $224 \times 224 \times 3$, is processed by EfficientNetV2-B0 to generate rich semantic features. These features are further enhanced through a series of custom convolutional layers, each followed by batch normalization to ensure training stability. The resulting feature maps are flattened and passed through a sequence of fully connected layers with dimensions $128 \rightarrow 128 \rightarrow 1024 \rightarrow 3136$, culminating in a classifier that predicts the authenticity of the input image.

### EfficientNetV2-B0 backbone

**Fig. 3:**
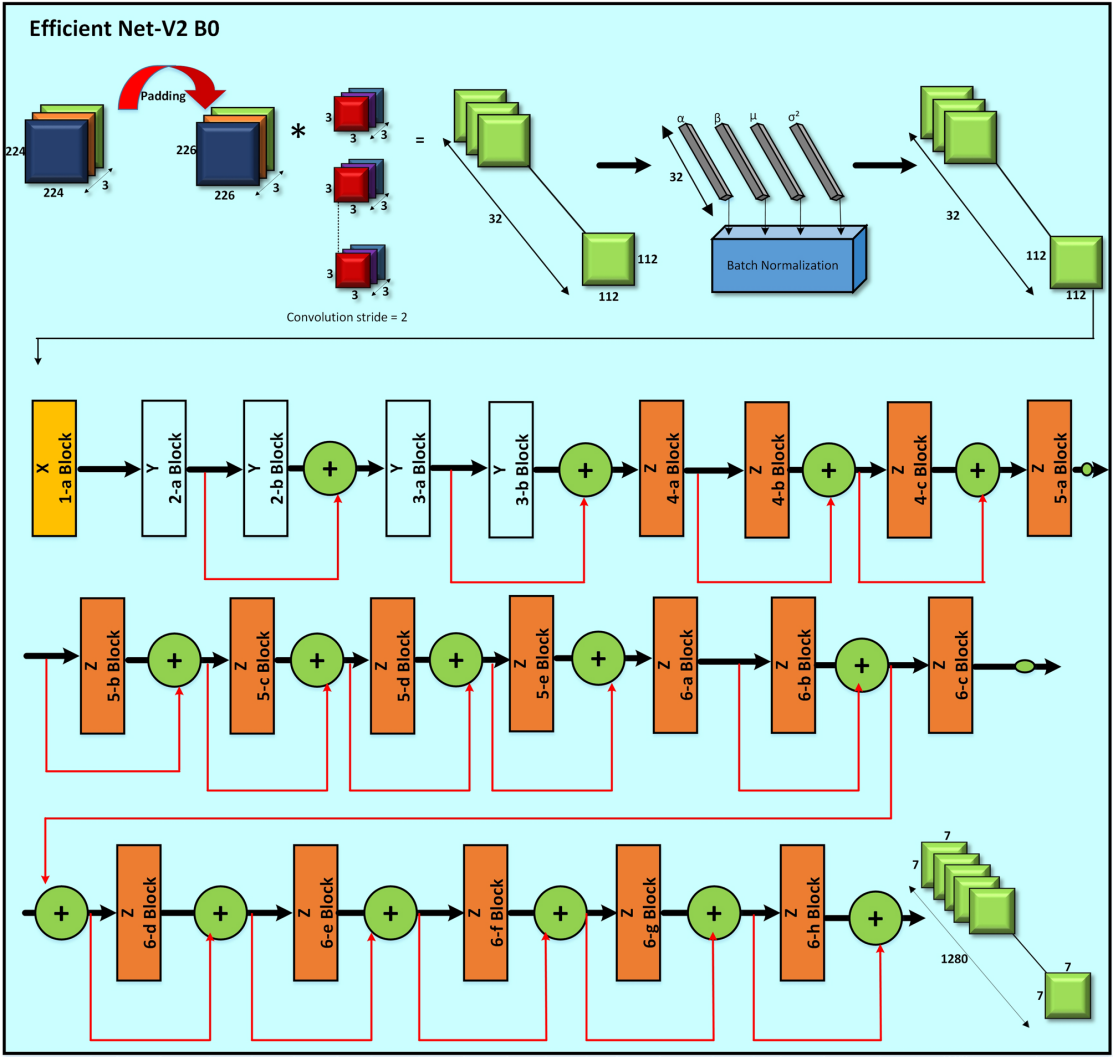
Model architecture of EfficientV2 B0 block.

EfficientNetV2-B0 (Fig. 3) serves as the core feature extractor, leveraging a compound scaling strategy that balances network depth, width, and resolution to optimize efficiency and performance. The architecture comprises three distinct sub-block types–X, Y, and Z–positioned at different stages of the network. These sub-blocks employ fused MBConv or depthwise separable convolutions, enhanced by residual connections to facilitate gradient flow and feature reuse. The X sub-block (1-a) initiates feature extraction, while Y sub-blocks (2-a, 2-b, 3-a, 3-b) handle early-stage feature refinement. The Z sub-blocks (4-a to 6-h) focus on extracting complex, abstract features in the deeper layers.

### X sub-block

**Fig. 4:**
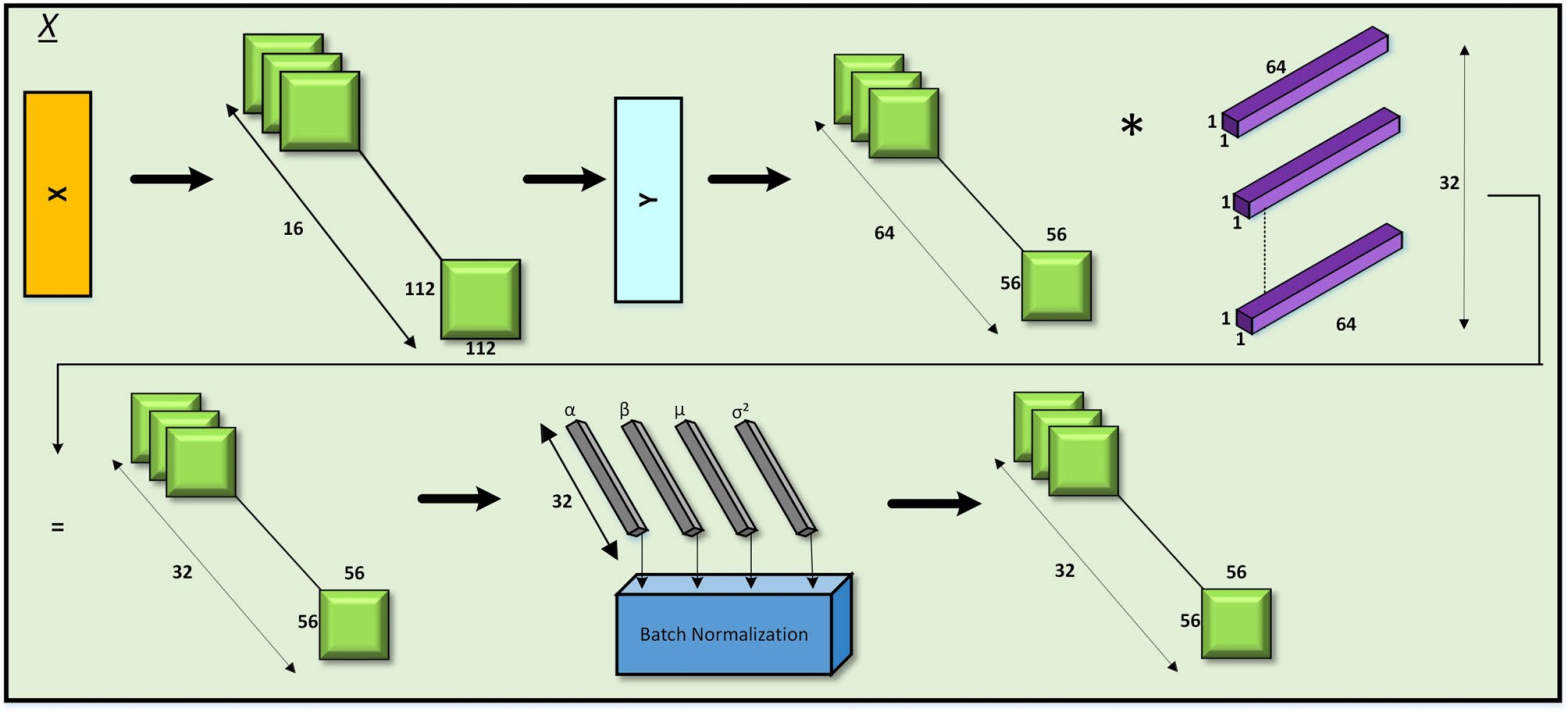
Model architecture of X block.

The X sub-block (1-a) (Fig. 4) processes a low-dimensional input feature map of $112 \times 112 \times 16$, applying a convolutional operation to downsample it to a high-dimensional feature map of $56 \times 56 \times 64$. This is achieved through pointwise convolutions, followed by batch normalization and a dimension reduction to 32 channels. This operation establishes a foundation for subsequent feature processing while maintaining computational efficiency.

### Y sub-blocks

**Fig. 5:**
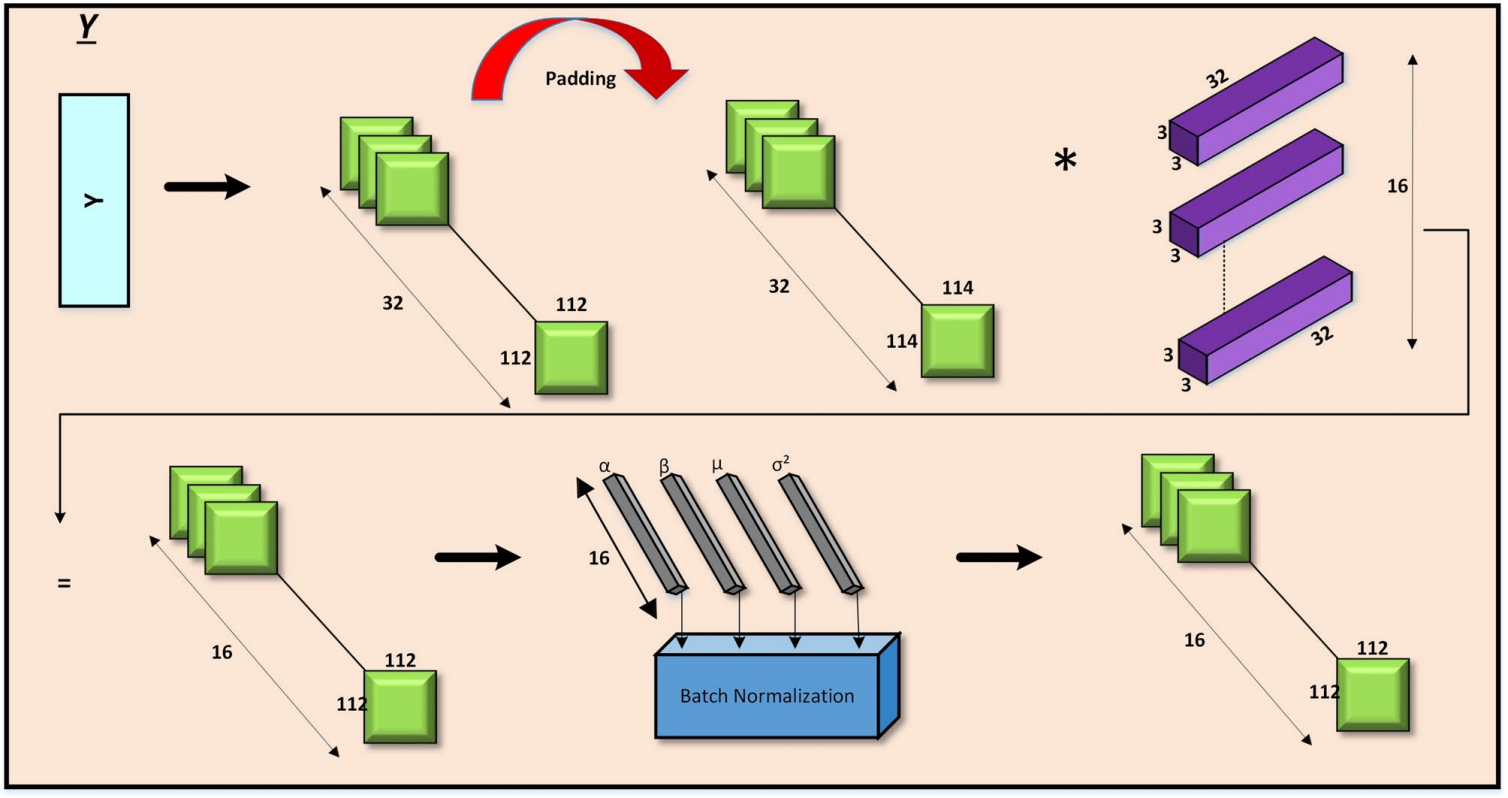
Model architecture of Y block.

The Y sub-blocks (2-a to 3-b) (Fig. 5) utilize padded $3 \times 3$ convolutions to preserve spatial dimensions and capture local patterns effectively. These layers progressively reduce the number of channels (e.g., from 32 to 16) in a structured manner, retaining critical high-resolution information. Shortcut connections and batch normalization are incorporated to enhance gradient stability and ensure seamless information flow across layers.

### Z sub-blocks

**Fig. 6:**
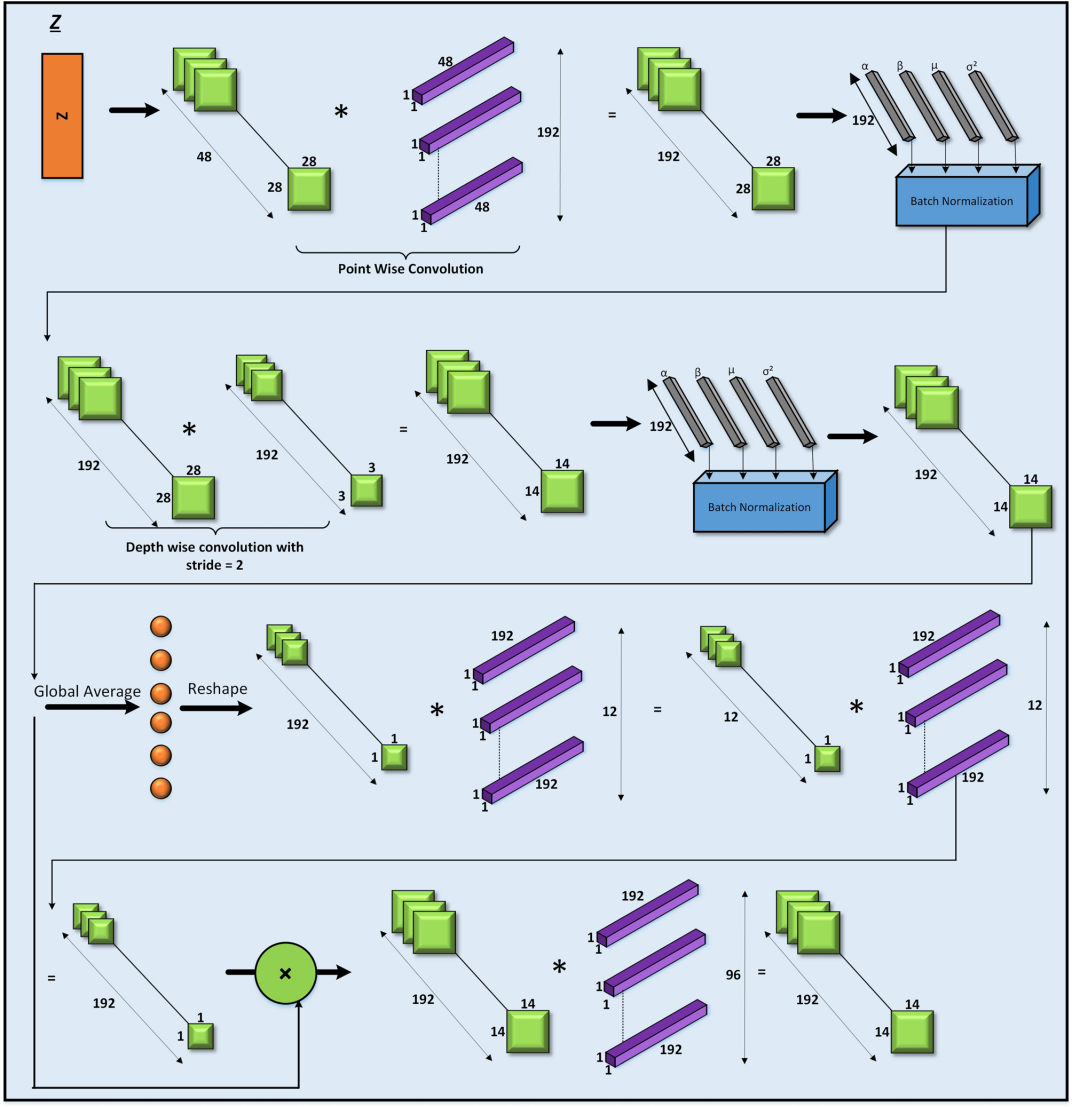
Model architecture of Z Block.

The Z sub-blocks (4-a to 6-h) (Fig. 6) are the deepest and most complex modules in the architecture. They begin with pointwise convolutions to expand feature dimensions (e.g., from 48 to 192), followed by depthwise convolutions with a stride of 2 to reduce spatial size. Squeeze-and-Excitation (SE) modules are integrated, using global average pooling to generate attention vectors that scale channel-wise responses. After gating and feature projection, residual connections combine the transformed and original signals, enabling the Z sub-blocks to construct robust, abstract features. This structure enhances the network’s efficiency and agility in detecting tampering artifacts.

### Feature mapping via convolutional function approximation

Let $\mathcal {X} \subset \mathbb {R}^{H \times W \times C}$ represent the input space of RGB images, where each image $\textbf{x}_i \in \mathcal {X}$ is of size $(H = 224, W = 224, C = 3)$. The deep convolutional feature extractor, a differentiable and deterministic map $\phi : \mathcal {X} \rightarrow \mathbb {R}^{d}$, transforms input images into high-dimensional latent vectors. Specifically in Eq. (1)

1 $$\begin{aligned} \textbf{z}_i = \phi (\textbf{x}_i; \theta _\phi ) \in \mathbb {R}^{d} \end{aligned}$$

with $d \approx 1280$. Here, $\textbf{z}_i$ denotes the deep feature representation of image $\textbf{x}_i$, and $\theta _\phi \in \mathbb {R}^{p}$ are the learnable parameters of the feature extractor, initialized from ImageNet pretraining and kept frozen during early training. This approach captures semantically rich, invariant properties, mitigating nuisance variables like background clutter, lighting, and geometric transformations.

### Compound scaling in EfficientNetV2

EfficientNetV2 is a CNN family designed with compound scaling, a systematic approach to balance network depth *d*, width *w*, and input resolution *r*. Rather than arbitrary or independent scaling, compound scaling enforces a multi-objective constraint to optimize these dimensions. Let $d \in \mathbb {N}$ denote the number of layers (depth), $w \in \mathbb {R}^+$ represent the number of channels per layer (width), and $r \in \mathbb {N}$ indicate the input resolution. The compound scaling method defined as shown in Eq. (2)

2 $$\begin{aligned} d = \alpha ^\phi d_0, w = \beta ^\phi w_0, r = \gamma ^\phi r_0 \end{aligned}$$

subject to the constraint as shown in Eq. (3)

3 $$\begin{aligned} \alpha \cdot \beta ^2 \cdot \gamma ^2 \approx \kappa \end{aligned}$$

where $\phi \in \mathbb {Z}^+$ is a compound coefficient controlling model size, $\alpha , \beta , \gamma > 0$ are constants derived via grid search or optimization, $\kappa \in \mathbb {R}^+$ limits computational resources (e.g., FLOPs), and $(d_0, w_0, r_0)$ are baseline parameters for $\phi = 0$. This ensures computational cost grows polynomially with $\phi$, enabling efficient and uniform scaling.

### Fused-MBConv and SE attention blocks

EfficientNetV2B0 incorporates two key architectural innovations: Fused-MBConv blocks in early layers and Mobile Inverted Bottleneck Convolutions (MBConv) in deeper layers. A Fused-MBConv block is defined as shown in Eq. (4)

4 $$\begin{aligned} \textbf{y} = \text {BN}(\text {Conv}_{3 \times 3}(\rho (\text {Conv}_{1 \times 1}(\textbf{x})))) \end{aligned}$$

while a standard MBConv block as shown in Eq. (5)

5 $$\begin{aligned} \textbf{y} = \text {BN}(\text {DWConv}_{3 \times 3}(\rho (\text {Expand}(\textbf{x})))) \end{aligned}$$

where $\text {BN}(\cdot )$ denotes batch normalization, $\rho (\cdot )$ is the Swish activation defined as shown in Eq. (6)

6 $$\begin{aligned} \rho (x) = x \cdot \text {sigmoid}(x) \end{aligned}$$

$\text {DWConv}_{3 \times 3}$ is a depthwise convolution, and $\text {Expand}(\cdot )$ is a channel-wise expansion operation. Additionally, Squeeze-and-Excitation (SE) attention modules reweight features by modeling inter-channel dependencies. For a feature map $\textbf{F} \in \mathbb {R}^{H \times W \times C}$, the SE operation is defined as shown in Eq. (7)

7 $$\begin{aligned} \textbf{s} = \sigma \left( W_2 \cdot \rho (W_1 \cdot \text {GAP}(\textbf{F}))\right) , \textbf{F}' = \textbf{F} \odot \textbf{s} \end{aligned}$$

where $\text {GAP}$ is global average pooling, $W_1 \in \mathbb {R}^{\frac{C}{r} \times C}$ and $W_2 \in \mathbb {R}^{C \times \frac{C}{r}}$ are fully connected weights, $\sigma (\cdot )$ is the sigmoid activation, and $\odot$ denotes element-wise multiplication for channel-wise scaling. This dynamic reweighting enhances salient features, such as tampered edges and blending artifacts, while suppressing irrelevant channels.

### Final feature representation

The EfficientNetV2 block outputs a high-dimensional tensor $\textbf{T} \in \mathbb {R}^{H' \times W' \times C'}$, with $H' = W' = 7$ and $C' \approx 1280$. This tensor is transformed into a 1D vector via global average pooling as shown in Eq. (8)

8 $$\begin{aligned} \textbf{z}_i = \frac{1}{H'W'} \sum _{h=1}^{H'} \sum _{w=1}^{W'} \textbf{T}_{h,w,:} \end{aligned}$$

yielding $\textbf{z}_i \in \mathbb {R}^{1280}$, a compact descriptor capturing high-level semantics, such as object composition and color homogeneity, while filtering out low-level noise.

### Transfer learning justification

The convolutional weights $\theta _\phi$ are initialized with a pretrained ImageNet model, leveraging prior knowledge of natural image statistics as shown in Eq. (9)

9 $$\begin{aligned} \theta _\phi = \arg \min _\theta \mathbb {E}_{(\textbf{x}, y) \sim \mathcal {D}_{\text {ImageNet}}} \left[ \mathcal {L}_{\text {CE}}(y, f_\theta (\textbf{x})) \right] \end{aligned}$$

where $\mathcal {L}_{\text {CE}}$ is the cross-entropy loss. This accelerates convergence and enhances generalization, especially for limited tampered image datasets. Early layers of $\phi (\cdot )$ capture general patterns like edges, textures, and blobs, and freezing them prevents overfitting and ensures stability during initial training.

### Regularized deep classification head and optimization with focal loss

The high-dimensional feature vector $\textbf{z}_i \in \mathbb {R}^{d}$ extracted by the EfficientNetV2B0 backbone is processed by a deep classification head $h(\cdot )$, comprising two fully connected hidden layers with ReLU activations, dropout regularization, and a final sigmoid classifier. The transformation is defined as shown in Eq. (10)

10 $$\begin{aligned} h(\textbf{z}_i) = \sigma \left( {W}_3^\top \rho \left( {W}_2 \rho \left( {W}_1 \cdot \text {Flatten}(\textbf{z}_i) + {b}_1 \right) + {b}_2 \right) + b_3 \right) \end{aligned}$$

11 $$\begin{aligned} \tilde{h}(\textbf{z}_i) = \sigma \left( \textbf{w}_3^\top \cdot \text {Dropout}(h(\textbf{z}_i)) + b_3 \right) \end{aligned}$$

12 $$\begin{aligned} \Vert \textbf{W}_i\Vert _2^2 \le \lambda \end{aligned}$$

where $\rho (x) = \max (0, x)$ is ReLU, $\sigma (z) = \frac{1}{1 + e^{-z}}$ is sigmoid, ${W}_1 \in \mathbb {R}^{d \times 1024}$, ${W}_2 \in \mathbb {R}^{1024 \times 128}$, ${W}_3 \in \mathbb {R}^{128}$, and $\textbf{b}_1$, $\textbf{b}_2$, $\textbf{b}_3$ are biases. Dropout with rate $p = 0.5$ is applied before the final layer as shown in Eq. (11) Weights are constrained by $\ell _2$-norm as shown in Eq. (12) to prevent overfitting.

To address class imbalance between authentic and tampered images, Focal Loss modifies binary cross-entropy to focus on hard examples as shown in Eq. (13)

13 $$\begin{aligned} \mathcal {L}_{\text {focal}}(y_i, \hat{p}_i) = -\alpha y_i (1 - \hat{p}_i)^{\gamma } \log (\hat{p}_i) - (1 - \alpha )(1 - y_i) \hat{p}_i^{\gamma } \log (1 - \hat{p}_i) \end{aligned}$$

14 $$\begin{aligned} \mathcal {J}(\theta ) = \frac{1}{N} \sum _{i=1}^N \mathcal {L}_{\text {focal}}(y_i, f_\theta (\textbf{x}_i)) + \lambda \sum _j \Vert \theta _j\Vert _2^2 \end{aligned}$$

where $\alpha = 0.25$ balances classes, and $\gamma = 2.0$ is the focusing parameter. The total objective is shown in Eq. (14) optimized using Adam with moment updates are shown in Eq. (15)

15 $$\begin{aligned} \theta _t \leftarrow \theta _{t-1} - \eta \cdot \frac{m_t}{\sqrt{v_t} + \epsilon } \end{aligned}$$

16 $$\begin{aligned} m_t = \beta _1 m_{t-1} + (1 - \beta _1) \nabla _\theta \mathcal {J}(\theta ) \end{aligned}$$

17 $$\begin{aligned} v_t = \beta _2 v_{t-1} + (1 - \beta _2) \Vert \nabla _\theta \mathcal {J}(\theta )\Vert ^2 \end{aligned}$$

where mt and vt are defined as shown in Eqs. (16) and (17) with $\beta _1 = 0.9$, $\beta _2 = 0.999$, $\epsilon = 10^{-8}$, and $\eta = 10^{-3}$. A ReduceLROnPlateau strategy halves $\eta$ if validation AUC plateaus, ensuring convergence stability.

## Results and discussion

The proposed CNN + EfficientNetV2B0 model was evaluated on four benchmark image forgery datasets-CASIA v1, Defacto (Splicing), Columbia, and MICC-F2000-covering diverse resolutions, compression schemes, and manipulation types. Across metrics like AUC, accuracy, F1-score, precision, and recall, the model demonstrated consistently high performance and generalizability. The following sections provide a detailed dataset-wise analysis with technical explanations of the results.

### Analysis on CASIA v1 dataset

**Fig. 7:**
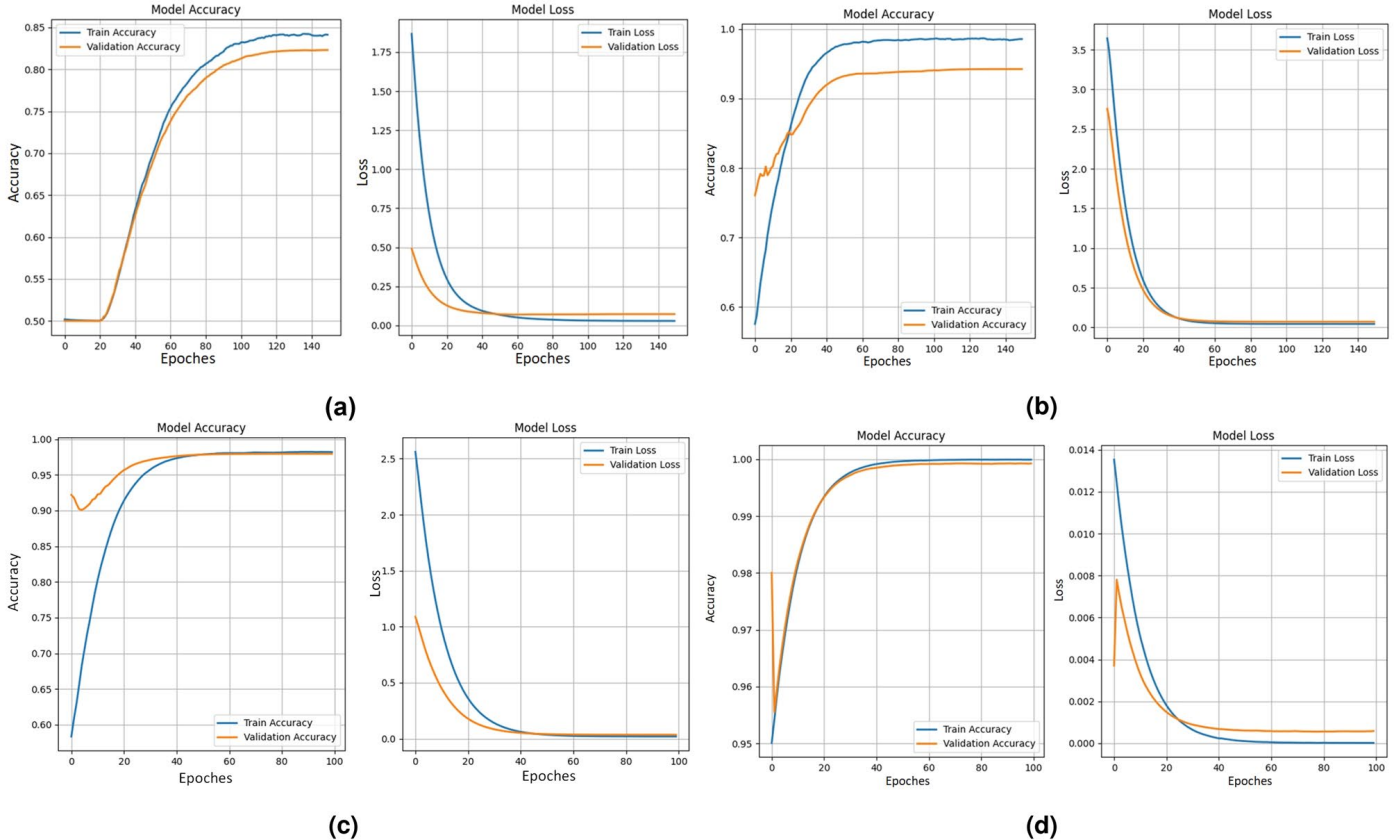
Training and validation of model accuracy and model loss graph obtained for (**a**) CASIA v1 dataset (**b**) Columbia dataset (**c**) MICC-F2000 dataset (**d**) DEFACTO Splicing dataset by proposed model.

The proposed model achieved an accuracy of 83.76% as shown in Fig. 7a and an AUC of 0.9131 as shown in Fig. 10a on the CASIA v1 dataset, a commendable performance given the dataset’s inherent challenges. CASIA v1 comprises JPEG-compressed images at low resolutions ( 384$\times$256), which introduce compression artifacts that obscure subtle tampering cues like texture discontinuities or boundary incoherencies. These artifacts hinder the EfficientNetV2B0 backbone’s ability to extract pristine features, making classification more difficult. The dataset’s focus on splicing manipulations, often with crude blending, further increases the risk of overfitting due to the variability in tampered content.Despite these challenges, the model demonstrated strong generalizability, as evidenced by a precision of 0.8367 and a recall of 0.8391, both shown in Fig. 9a , supported by Focal Loss, which emphasizes hard-to-classify samples, and SE attention blocks that amplify informative features. The corresponding F1 score of 0.8349, also in Fig. 9a , indicates a reliable trade-off between precision and recall, affirming the model’s robustness in handling noisy, low-quality data.

**Fig. 8:**
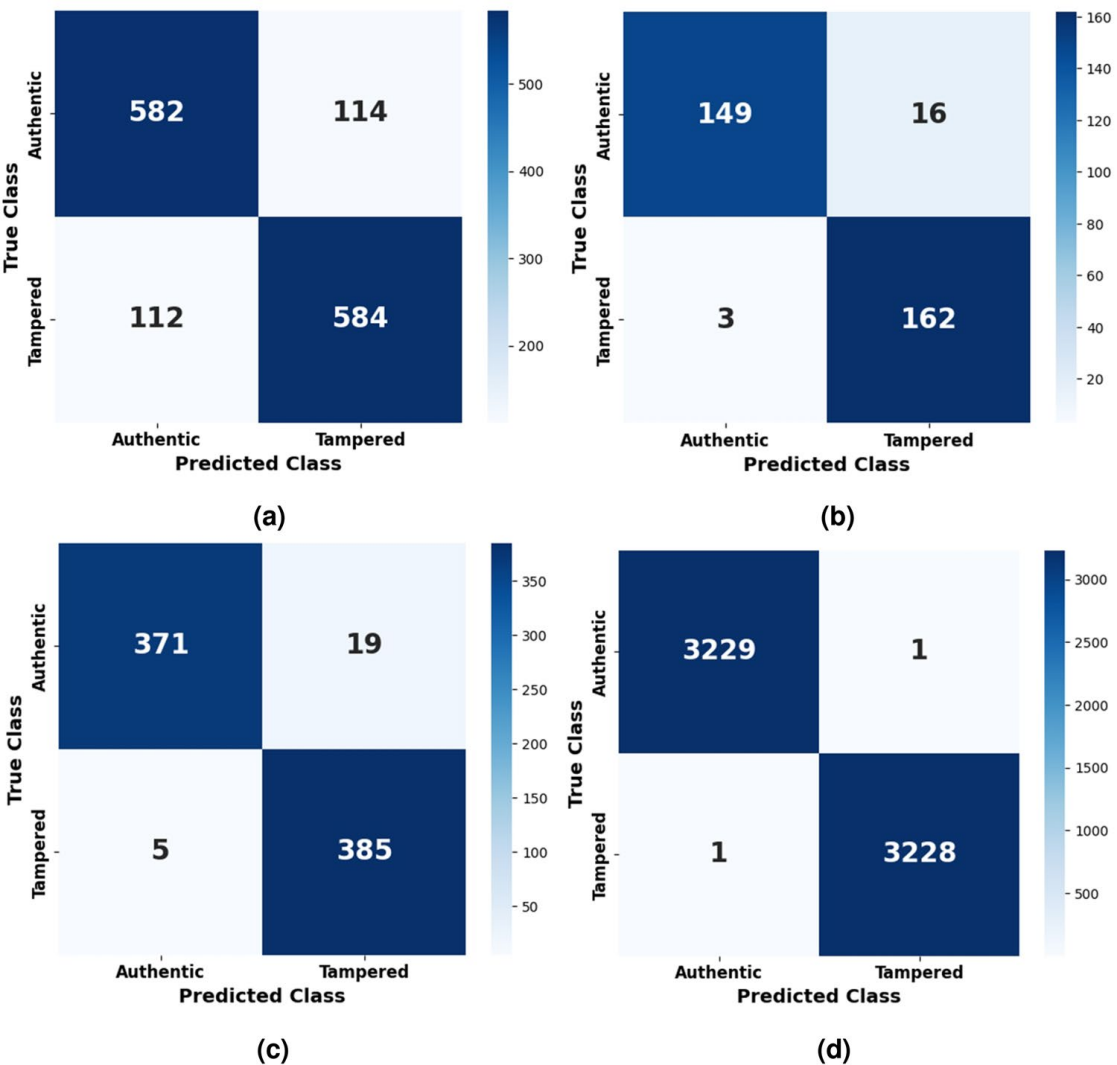
Confusion matrix obtained for (**a**) CASIA v1 dataset (**b**) Columbia dataset (**c**) MICC-F2000 dataset (**d**) DEFACTO Splicing dataset by proposed model.

The confusion matrix for CASIA v1, depicted in Fig. 8a , offers deeper insight into the model’s classification behavior. It shows a balanced distribution of true positives (TP) and true negatives (TN), reflecting the model’s ability to correctly identify both tampered and authentic images. However, a modest number of false positives (FP) and false negatives (FN) are also observed, which is expected given the dataset’s low resolution and compression artifacts. The false positives may arise when authentic images exhibit patterns or textures that resemble tampered artifacts, while false negatives can occur when tampered regions are too subtle or heavily blended. Nevertheless, the confusion matrix highlights a strong overall classification performance, validating the effectiveness of the proposed architecture in handling real-world forgery detection scenarios.

### Analysis on defacto (splicing) dataset

**Fig. 9:**
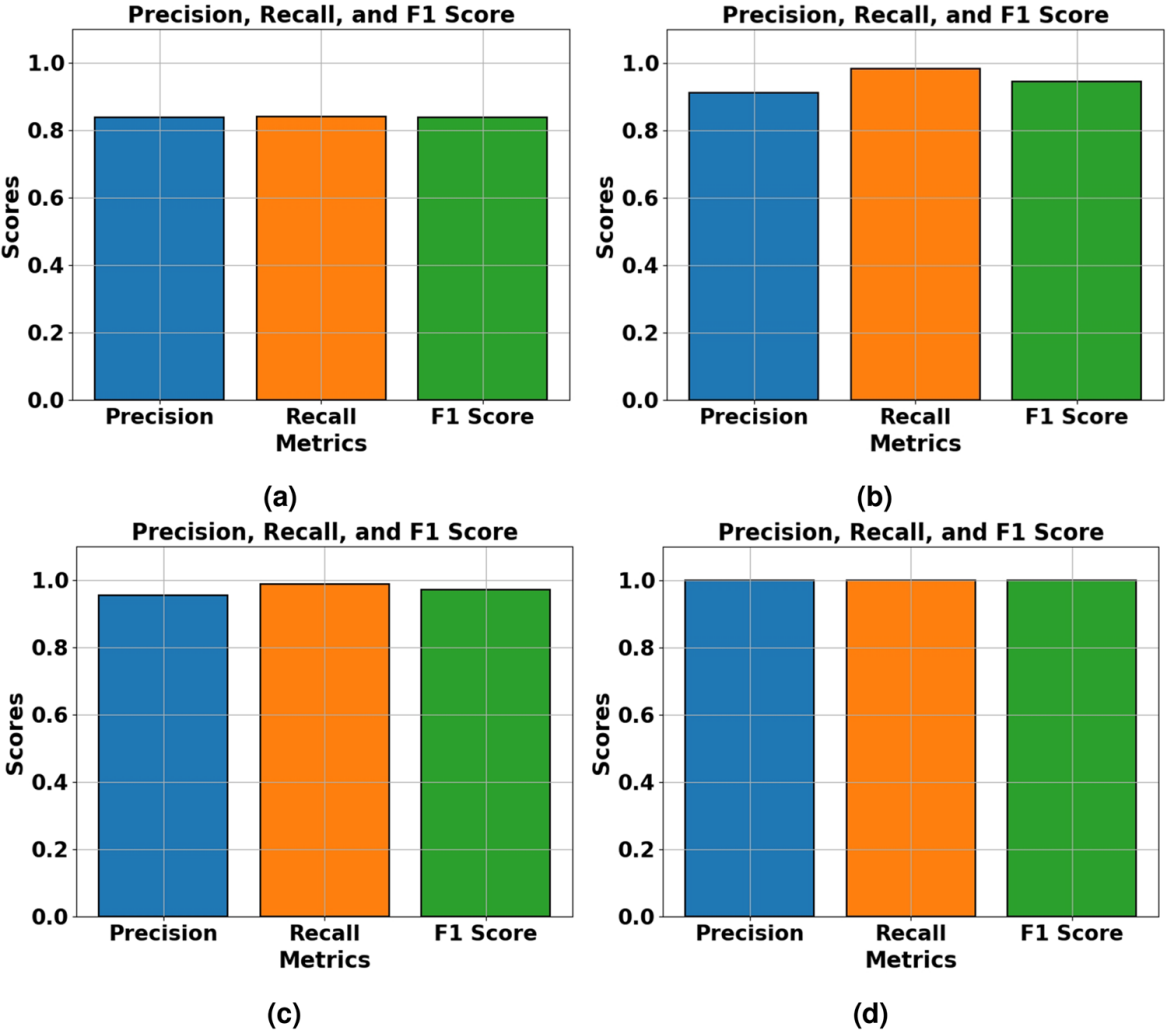
Precision, Recall and F1 score obtained for (**a**) CASIA v1 dataset (**b**) Columbia dataset (**c**) MICC-F2000 dataset (**d**) DEFACTO Splicing dataset by proposed model.

**Fig. 10:**
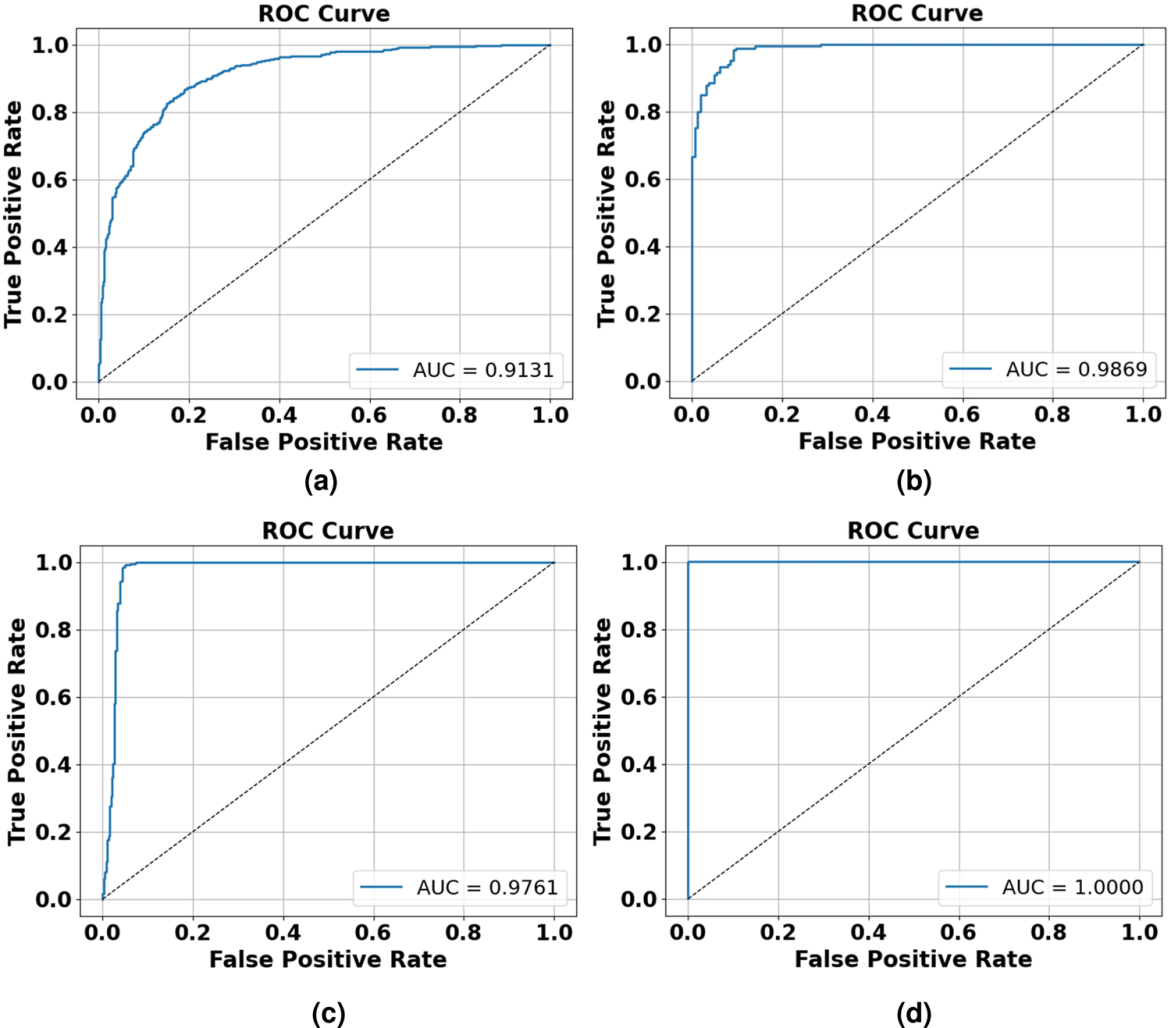
ROC curves obtained for (**a**) CASIA v1 dataset (**b**) Columbia dataset (**c**) MICC-F2000 dataset (**d**) DEFACTO Splicing dataset by proposed model.

On the Defacto (Splicing) dataset, the model delivered near-perfect performance, achieving an AUC of 1.0000 as shown in Fig. 10d , accuracy of 99.97% as shown in Fig. 7d , and identical precision, recall, and F1 scores of 0.9997 as shown in Fig. 9d . This exceptional performance stems from favorable dataset characteristics. Defacto’s high-resolution PNG images, free of compression artifacts, enhance the feature extractor’s ability to detect distinct manipulation boundaries and subtle variations. The dataset’s exclusive focus on splicing allows the model to specialize in identifying spatial disturbances introduced by external content. Fused-MBConv blocks preserve fine-grained structural details, while SE attention mechanisms reweight feature maps toward tampered regions, making the model highly effective in contextual tampering scenarios.

The confusion matrix shown in Fig. 8d further confirms the model’s outstanding accuracy. It reveals an almost perfect diagonal dominance, with virtually all authentic and tampered images correctly classified, resulting in a negligible number of false positives (FP) and false negatives (FN). This indicates that the model rarely mislabels genuine images as tampered, nor does it miss tampered cases-a critical requirement for real-world forensic applications. The extremely low error rate depicted in the confusion matrix reflects the synergy between the EfficientNetV2B0 backbone, which captures high-level semantic features, and the regularized CNN classification head, which ensures fine-grained decision boundaries. Together, they enable the model to achieve consistent, robust, and interpretable classification results under ideal imaging conditions.

### Analysis on columbia dataset

The model achieved a remarkable 94.24% accuracy as shown in Fig. 7b and an AUC of 0.9869 as shown in Fig. 10b on the Columbia dataset, demonstrating strong generalization to uncompressed, high-quality images. Columbia’s TIFF format preserves pixel-level details, enabling the EfficientNetV2B0 backbone to detect splicing-induced abnormalities without interference from compression noise. The recall of 0.9818 indicates excellent sensitivity to tampered samples, which is critical for forensic applications where missed detections could have serious consequences. However, the slightly lower precision of 0.9101 reflects a few instances where genuine images with complex textures were mistakenly flagged as tampered, likely due to overlapping statistical cues. The consistently high F1 score (0.9446) and AUC confirm robust discriminative performance, emphasizing the role of global average pooling and high-level semantic descriptors in helping the classification head define precise decision boundaries, as illustrated in Fig. 9b.

The confusion matrix for the Columbia dataset, shown in Fig. 8b , offers further evidence of the model’s effectiveness. It shows a high number of true positives (TP) and true negatives (TN), with only a minor presence of false positives (FP)–cases where authentic images were incorrectly labeled as tampered–and very few false negatives (FN). These misclassifications are expected in datasets containing rich texture or patterned content that may resemble forgery artifacts. Nevertheless, the matrix highlights the model’s strong ability to separate authentic and manipulated images, confirming its robustness when dealing with uncompressed images that retain fine-grained structural information. This level of performance suggests the model is well-suited for practical forensic environments where data quality is high and detection accuracy is paramount.

### Analysis on MICC-F2000 dataset

The model exhibited excellent performance on the MICC-F2000 dataset, achieving 96.92% accuracy as shown in Fig. 7c , an AUC of 0.9761 as shown in Fig. 10c , and an F1 score of 0.9698 as shown in Fig. 9c . This dataset features high-resolution JPEG images with diverse manipulations, including copy-move and splicing, making it a suitable benchmark for evaluating the model’s robustness. The model’s generalizability stems from its modular architecture, where the ImageNet-pretrained EfficientNetV2B0 backbone extracts deep structural features, while the CNN classification head applies non-linear transformations to fine-tune classification sensitivity. A recall of 0.9872, shown in Fig. 9c , reflects the model’s near-perfect ability to detect tampered images, which is critical for security-sensitive applications. The precision of 0.9530, also in Fig. 9c , indicates a low false-positive rate, aided by dropout regularization and L2 weight constraints, which help maintain stable and accurate decision boundaries. Moreover, oversampling and the application of Focal Loss successfully addressed the dataset’s limited size and class imbalance, further validating the model’s scalability and adaptability in handling complex, high-variance forgery scenarios.

The confusion matrix for MICC-F2000, illustrated in Fig. 8c , provides additional confirmation of the model’s discriminative capability. It displays a dominant diagonal, signifying a high number of correctly classified authentic and tampered images, and a minimal presence of false positives and false negatives. The few misclassifications are likely due to subtle manipulation traces or background inconsistencies that resemble authentic patterns. Nevertheless, the overall distribution in the confusion matrix reinforces the model’s consistent classification accuracy across varied manipulation types and high-resolution content. This performance demonstrates the model’s effectiveness in real-world forensic environments, where detecting a wide spectrum of tampering techniques with high reliability is essential.

## Qualitative and quantitative analysis

The EfficientNetV2B0-based CNN model is tested on CASIA v1, Defacto, MICC-F2000, and Columbia datasets that reflect varied image types and forgery models. The datasets are challenged in different ways, ranging from noisy JPEGs to high-resolution TIFFs that call for accurate tamper detection. AUC, F1-score, accuracy, precision, and recall are used to measure performance to guarantee high-level forensic applicability. The high-level feature extraction and Focal Loss of the model provide better performance, discussed in the subsequent comparative analysis.

### Comparative analysis of CASIA v1 dataset

The CASIA v1 dataset, with its spliced, compressed, low-resolution images, poses significant challenges due to noisy artifacts. Models like CANF (AUC: 0.91, F1: 0.73)^23^, CCLHF-Net (AUC: 0.84, F1: 0.75)^28^, ConvNeXtFF (AUC: 0.85, F1: 0.58)^15^, and CR-CNN (AUC: 0.78, F1: 0.47)^29^ exhibit varied performance. Other models, such as MVSS-Net++ (F1: 0.77)^48^, IML-ViT (F1: 0.81)^39^, and MSF-Net (AUC: 0.90, F1: 0.63)^47^, perform reasonably well. The proposed model surpasses these with an AUC of 0.9131, F1 of 0.8379, accuracy of 83.76%, precision of 83.67%, and recall of 83.91% as shown in Table 3. While CANF matches closely in AUC, its lower F1 indicates less balanced performance. Many models struggle with recall (e.g., ISIE-Net, F1: 0.53) or lack comprehensive metrics. The proposed model’s strength lies in EfficientNetV2B0’s scalable feature extraction and Focal Loss, enabling robust learning from complex CASIA samples.

**Table 3:** Performance comparison on CASIA v1 dataset.

Dataset: CASIA v1							
Ref. No.	Model	Year	AUC	F1 score	Accuracy	Precision	Recall
^23^	CANF	2024	0.91	0.73	–	–	–
^28^	CCLHF-Net	2024	0.84	0.75	–	–	–
^15^	ConvNeXtFF	2023	0.85	0.58	–	–	–
^29^	CR-CNN	2020	0.78	0.47	–	–	–
^30^	DMDC-Net	2023	–	0.27	–	–	–
^31^	EITL-Net	2024	–	0.55	–	–	–
^32^	EMT-Net	2023	0.85	0.45	–	–	–
^33^	EXIF-SC	2020	–	0.61	–	–	–
^34^	FCN	2021	0.79	0.77	–	–	–
^35^	GSC-Net	2020	0.83	0.47	–	–	–
^36^	GSR-Net	2020	–	0.57	–	–	–
^37^	HiFi-Net	2023	–	0.09	–	–	–
^38^	IF-OSN	2022	0.87	0.5	–	–	–
^39^	IML-ViT	2024	–	0.81	–	–	–
^40^	ISIE-Net	2025	0.82	0.53	–	–	–
^41^	locate-Net	2022	0.75	0.27	–	–	–
^42^	Loma	2025	–	0.76	–	–	–
^43^	M2BG	2024	–	0.65	–	–	–
^44^	MAPS-Net	2024	0.8	0.55	–	–	–
^45^	MPC	2025	–	0.74	–	–	–
^46^	MSFF	2022	0.7	0.45	–	–	–
^47^	MSF-Net	2025	0.9	0.63	–	–	–
^48^	MVSS-Net++	2022	–	0.77	–	–	–
^49^	NCL	2023	0.86	0.59	–	–	–
^50^	NCNS	2023	0.85	0.66	–	–	–
^51^	OSN	2022	0.87	0.5	–	–	–
^52^	PCL	2023	0.75	0.46	–	–	–
^53^	PSCC-NEt	2023	0.87	0.55	–	–	–
^54^	R-CNN+Mobile Net	2022	–	0.64	–	0.61	0.68
^46^	RRA-Net	2022	–	0.7	–	–	–
^55^	SPAN	2020	0.83	0.38	–	–	–
^56^	SparseViT	2025	–	0.81	–	–	–
^57^	TB-Net	2023	–	0.59	–	–	–
^58^	TDA-Net	2021	0.83	0.58	–	–	–
^59^	TransForensics	2021	0.83	0.62	–	–	–
^60^	TruFOR	2023	–	0.69	–	–	–
^61^	VSS+SS2D	2025	–	0.72	–	–	–
Proposed	CNN+EfficientNetV2B0	2025	0.9131	0.8379	0.8376	0.8367	0.8391

### Comparative analysis of defacto (splicing) dataset

The Defacto dataset, featuring clean PNG splicing, is ideal for assessing semantic inconsistencies. Models like CAT-Net and the DRRU-Net^25^ family achieve high accuracy (0.988–0.99) but lack precision, recall, and F1 scores. In contrast, the proposed model achieves the highest accuracy (0.9997), AUC (1.0000), and identical F1, precision, and recall (0.9997) as shown in Table 4. SE blocks enhance focus on tampered regions, and Focal Loss ensures robustness on challenging samples. The EfficientNetV2B0 backbone and deep classification head enable precise splicing detection, demonstrating superior semantic and spatial perception.

**Table 4:** Performance comparison on defacto (splicing) dataset.

Dataset: defacto (splicing)							
Ref. No.	Model	Year	AUC	F1 score	Accuracy	Precision	Recall
^25^	CAT-Net	2023	–	–	0.99	–	–
^25^	DRRU-Net	2023	–	–	0.99	–	–
^25^	DRRU-Net LR	2023	–	–	0.99	–	–
^25^	DRRU-Net TL	2023	–	–	0.99	–	–
^25^	RRU-Net	2023	–	–	0.988	–	–
Proposed	CNN+EfficientNetV2B0	2025	1.0	0.9997	0.9997	0.9997	0.9997

### Comparative analysis of MICC-F2000 dataset

MICC-F2000’s high-resolution images and varied forgeries present detection challenges. Traditional methods like VGG19-SVM and ResNet50-SVM^24^ achieve 0.88 F1 and accuracy, while DCT+LBP+SVM^62^ reaches 0.94 accuracy and 0.92 precision. Hybrid approaches like ELA+VGG19^62^ show uneven performance. The proposed model outperforms these with an AUC of 0.9761, F1 of 0.9698, accuracy of 96.92%, precision of 95.30%, and recall of 98.72% as shown in Table 5 . Its high recall is critical for security applications. Leveraging EfficientNet’s capabilities and an optimized classification head, the model ensures better class separability and consistency than earlier approaches, which often sacrifice recall for accuracy.

**Table 5:** Performance comparison on MICC-F2000 dataset.

Dataset: MICC-F2000							
Ref. No.	Model	Year	AUC	F1 score	Accuracy	Precision	Recall
^24^	VGG19-SVM	2024	–	0.88	0.88	0.9	0.88
^24^	ResNet50-SVM	2024	–	0.88	0.88	0.89	0.88
^62^	DCT+SVM	2023	–	–	0.93	0.88	0.9
^62^	DCT+LBP+SVM	2023	–	–	0.94	0.92	0.89
^62^	ELA+VGG16	2023	–	–	0.89	0.88	0.9
^62^	ELA+VGG19	2023	–	–	0.9	0.89	0.88
^62^	ELA+RESNET50	2023	–	–	0.76	0.65	0.8
Proposed	CNN+EfficientNetV2B0	2025	0.9761	0.9698	0.9692	0.953	0.9872

### Comparative analysis of Columbia dataset

The Columbia dataset’s uncompressed TIFF images demand precise localization. Competitive models include ConvNeXtFF (AUC: 0.93, F1: 0.88)^15^, DMDC-Net (F1: 0.80, Precision: 0.91)^30^, MSFF (AUC: 0.96, F1: 0.83)^46^, IML-ViT (F1: 0.91)^39^, and ViT-132 (F1: 0.90)^11^. Others, like SPAN^55^, MAPS-Net^44^, and TDA-Net^58^, have lower F1 scores (0.81, 0.74, 0.73). The proposed model achieves an AUC of 0.9869, F1 of 0.9446, accuracy of 94.24%, precision of 91.01%, and recall of 98.18% as shown in Table 6, balancing metrics effectively. SE attention and global average pooling enable accurate tamper localization and semantic separation, essential for forensics.

**Table 6:** Performance comparison on Columbia dataset.

Dataset: Columbia							
Ref. No.	Model	Year	AUC	F1 score	Accuracy	Precision	Recall
^26^	CAT-Net	2022	–	0.79	–	–	–
^15^	ConvNeXtFF	2023	0.93	0.88	–	–	–
^30^	DMDC-Net	2023	–	0.8	–	0.91	0.7
^31^	EITL-Net	2024	–	0.87	–	–	–
^12^	FCN	2022	–	0.53	–	–	–
^63^	Forensic-Net	2023	–	0.63	–	0.65	0.62
^37^	HiFi-Net	2023	–	0.83	–	–	–
^38^	IF-OSN	2022	0.86	0.7	–	–	–
^39^	IML-ViT	2024	–	0.91	–	–	–
^40^	ISIE-Net	2025	0.89	0.71	–	–	–
^42^	Loma	2025	–	0.88	–	–	–
^44^	MAPS-Net	2024	0.89	0.74	–	–	–
^45^	MPC	2025	–	0.94	–	–	–
^46^	MSFF	2022	0.96	0.83	–	–	–
^47^	MSF-Net	2025	–	0.78	–	–	–
^49^	NCL	2023	0.92	0.8	–	–	–
^51^	OSN	2022	0.86	0.7	–	–	–
^52^	PCL	2023	0.76	0.69	–	–	–
^46^	RRA-Net	2022	–	0.75	–	–	–
^60^	TruFOR	2023	–	0.79	–	–	–
^11^	ViT-132	2025	–	0.9	–	0.89	0.91
^61^	VVS+SS2D	2025	–	0.94	–	–	–
^64^	TPB-Net	2024	0.89	0.7	–	–	–
^65^	C2R-Net	2020	–	0.69	–	0.8	0.61
^29^	CR-CNN	2020	–	0.43	–	–	–
^66^	CAT-NET	2021	–	0.55	–	–	–
^67^	DFCN	2021	0.62	0.41	–	–	–
^33^	EXIF-SC	2020	–	0.51	–	–	–
^36^	GSR-Net	2020	–	0.61	–	–	–
^68^	Noiseprint	2020	0.84	0.36	–	–	–
^69^	RTAG	2021	–	0.73	–	0.62	0.89
^55^	SPAN	2020	0.93	0.81	–	–	–
^55^	SPAN	2020	–	0.48	–	–	–
^58^	TDA-Net	2021	0.89	0.73	–	–	–
Proposed	CNN+EfficientNetV2B0	2025	0.9869	0.9446	0.9424	0.9101	0.9818

### Summary of model comparison

Across CASIA v1, Defacto, MICC-F2000, and Columbia, the proposed EfficientNetV2B0-based CNN consistently outperforms CNN and transformer models, achieving optimal AUC and F1 scores. Unlike many prior approaches, it provides comprehensive metrics, ensuring transparency. The model excels across formats (JPEG, PNG, TIFF) and forgery types (splicing, copy-move, hybrid), driven by its pretrained backbone, deep classification head, and Focal Loss, delivering robust generalization and state-of-the-art performance in diverse forensic applications.The proposed model excels in detecting various manipulation types, including splicing, copy-move, and hybrid manipulations, across four benchmark datasets (CASIA v1, Defacto, MICC-F2000, Columbia). It demonstrates superior performance in splicing detection, gaining the highest AUC and F1 of 0.9997 on the Defacto dataset, outperforming models like DRRU-Net and CAT-Net. It also offers exceptional robustness to copy-move and hybrid forgeries, as reflected by an F1 score of 0.9698 and Recall of 0.9872 on the MICC-F2000 dataset. Furthermore, despite challenges such as compression and low resolution in the CASIA v1 dataset, the model achieves AUC = 0.9131 and F1 = 0.8379, outperforming models like MSF-Net and ConvNeXtFF in low-resolution scenarios. Concerning the real-time detection, the model’s usage of the computationally efficient EfficientNetV2B0 backbone, connected with a lightweight CNN classification head, provides minimal latency while retaining high accuracy. Unlike models that need expensive pixel-level annotations, our model achieves image-level classification, making it more suited for real-time deployment. The inclusion of Fused-MBConv blocks and SE attention allows the model to achieve fast convergence and decrease repetitive computations. Further, the use of transfer learning with frozen base layers greatly improves training efficiency, making the model scalable for real-world forensic systems that need frequent retraining across new datasets or tampering types. With invariably high F1-scores (>0.83) and AUC (>0.91) across all datasets, our model offers both stable performance and fast inference, making it ideal for practical deployment under constrained runtime conditions.

## Conclusion

This study introduces a strong image classification system for tampered image detection that surpasses the limitations of existing models in terms of accuracy and adaptability. Building on EfficientNetV2B0 as the base model, novel scaling and attention strategies, and training with Focal Loss to handle imbalanced class distributions, the model performs remarkably well on four benchmark datasets. The system not only outperforms both traditional and deep learning models in key metrics such as AUC, F1-score, precision, and recall, but also continues to perform robustly across diverse datasets and manipulation complexities. It stands out by outperforming 42 other models on CASIA v1 alone, and classifying images near-perfectly on Defacto and MICC-F2000. These results confirm that the proposed model can be a useful and adaptable solution for real-world applications in image forgery detection, with both reliability and flexibility.

## Data Availability

The datasets supporting the findings of this study are publicly available and have been appropriately cited within the manuscript. Additional data or clarifications can be obtained from the corresponding author upon reasonable request.

## References

[1] Li, Q., Wang, C., Zhou, X. & Qin, Z. Image copy-move forgery detection and localization based on super-bpd segmentation and dcnn. *Sc. Rep.* **12**, 14987 (2022). 36056097 10.1038/s41598-022-19325-y PMC9440200

[2] Shan, W., Yue, J., Ding, S. X. & Qiu, J. Mscscc-net: Multi-scale contextual spatial-channel correlation network for forgery detection and localization of jpeg-compressed image. *Sci. Rep.* **15**, 12509 (2025). 40216965 10.1038/s41598-025-97555-6 PMC11992245

[3] Alahmadi, A. et al. Passive detection of image forgery using dct and local binary pattern. *Signal Image Video Process.* **11**, 81–88 (2017).

[4] Isaac, M. M. & Wilscy, M. Multiscale local gabor phase quantization for image forgery detection. *Multimed. Tools Appl.* **76**, 25851–25872 (2017).

[5] Shen, X., Shi, Z. & Chen, H. Splicing image forgery detection using textural features based on the grey level co-occurrence matrices. *IET Image Process.* **11**, 44–53 (2017).

[6] Kanwal, N., Girdhar, A., Kaur, L. & Bhullar, J. S. Digital image splicing detection technique using optimal threshold based local ternary pattern. *Multimed. Tools Appl.* **79**, 12829–12846 (2020).

[7] Dua, S., Singh, J. & Parthasarathy, H. Image forgery detection based on statistical features of block dct coefficients. *Procedia Comput. Sci.* **171**, 369–378 (2020).

[8] Abd El-Latif, E. I., Taha, A. & Zayed, H. H. A passive approach for detecting image splicing based on deep learning and wavelet transform. *Arab. J. Sci. Eng*. **45**, 3379–3386 (2020).

[9] Kaur, N. Hybrid image splicing detection: Integrating clahe, improved cnn, and svm for digital image forensics. *Expert Syst. Appl*. 126756 (2025).

[10] Khoje, S. & Shinde, S. Evaluation of ripplet transform as a texture characterization for iris recognition. *J. Inst. Eng. (India) Series B*. **104**, 369–380 (2023).

[11] Pawar, D., Gowda, R. & Chandra, K. Image forgery classification and localization through vision transformers. *Int. J. Multimed. Inform. Retrieval* **14**, 1–11 (2025).

[12] Li, Z., You, Q. & Sun, J. A novel deep learning architecture with multi-scale guided learning for image splicing localization. *Electronics* **11**, 1607 (2022).

[13] Hao, X., Shao, L., He, X. & Zheng, X. Me: Multi-task edge-enhanced for image forgery localization. In *Proceedings of the 2024 7th International Conference on Image and Graphics Processing*, 327–333 (2024).

[14] Salloum, R., Ren, Y. & Kuo, C.-C.J. Image splicing localization using a multi-task fully convolutional network (mfcn). *J. Visual Commun. Image Representation* **51**, 201–209 (2018).

[15] Zhu, H., Cao, G., Zhao, M., Tian, H. & Lin, W. Effective image tampering localization with multi-scale convnext feature fusion. *J. Visual Commun. Image Representation* **98**, 103981 (2024).

[16] Liang, P., Li, Z., Tu, H. & Zhao, H. Lbrt: Local-information-refined transformer for image copy-move forgery detection. *Sensors* **24**, 4143 (2024). 39000921 10.3390/s24134143 PMC11244022

[17] Zhang, L. *et al.* Rethinking image forgery detection and localization via regression perspective. *IEEE Trans. Emerg. Topics Comput. Intell*. (2025).

[18] Ren, R. et al. Multi-scale attention context-aware network for detection and localization of image splicing: Efficient and robust identification network. *Appl. Intelligence* **53**, 18219–18238 (2023).

[19] Xiang, Y. *et al.* Image manipulation localization using dual-shallow feature pyramid fusion and boundary contextual incoherence enhancement. *IEEE Trans. Emerg. Topics Comput. Intell*. (2024).

[20] Gao, Z. *et al.* Generic image manipulation localization through the lens of multi-scale spatial inconsistence. In *Proceedings of the 30th ACM International Conference on Multimedia*, 6146–6154 (2022).

[21] Xu, Y., Zheng, J., Ren, J. & Fang, A. Feature aggregation and region-aware learning for detection of splicing forgery. *IEEE Signal Process. Lett.* **31**, 696–700 (2024).

[22] Ren, R. *et al.* Esrnet: Efficient search and recognition network for image manipulation detection. *ACM Trans. Multimed. Comput. Commun. Appl. (TOMM)*. **18**, 1–23 (2022).

[23] Zhou, Y., Wang, H., Zeng, Q., Zhang, R. & Meng, S. A contribution-aware noise feature representation model for image manipulation localization. *Knowl.-Based Syst.* **298**, 111988 (2024).

[24] Ibrahim, Z. S. & Hasan, T. M. Copy-move image forgery detection using deep learning approaches. In *2024 First International Conference on Software, Systems and Information Technology (SSITCON)*, 1–6 (IEEE, 2024).

[25] Seo, Y. & Kook, J. Drru-net: Dct-coefficient-learning rru-net for detecting an image-splicing forgery. *Appl. Sci.* **13**, 2922 (2023).

[26] Kwon, M.-J., Nam, S.-H., Yu, I.-J., Lee, H.-K. & Kim, C. Learning jpeg compression artifacts for image manipulation detection and localization. *Int. J. Comput. Vision* **130**, 1875–1895 (2022).

[27] Kumar, T., Brennan, R., & Bendechache, M. A comprehensive survey and future directions. *IEEE Access, Image data augmentation approaches* (2024).

[28] Yadav, K. D. K., Kavati, I. & Cheruku, R. Cclhf-net: Constrained convolution layer and hybrid features-based skip connection network for image forgery detection. *Arab. J. Sci. Eng.* **50**, 825–834 (2025).

[29] Yang, C., Li, H., Lin, F., Jiang, B. & Zhao, H. Constrained r-cnn: A general image manipulation detection model. In *2020 IEEE International conference on multimedia and expo (ICME)*, 1–6 (IEEE, 2020).

[30] Zhang, J., Wang, H. & He, P. Dual-branch multi-scale densely connected network for image splicing detection and localization. *Signal Process. Image Commun.* **119**, 117045 (2023).

[31] Guo, K., Zhu, H. & Cao, G. Effective image tampering localization via enhanced transformer and co-attention fusion. In *ICASSP 2024-2024 IEEE International Conference on Acoustics, Speech and Signal Processing (ICASSP)*, 4895–4899 (IEEE, 2024).

[32] Lin, X. et al. Image manipulation detection by multiple tampering traces and edge artifact enhancement. *Pattern Recognit.* **133**, 109026 (2023).

[33] Rozsa, A., Zhong, Z. & Boult, T. E. Adversarial attack on deep learning-based splice localization. In *Proceedings of the IEEE/CVF Conference on Computer Vision and Pattern Recognition Workshops*, 648–649 (2020).

[34] Chen, X., Dong, C., Ji, J., Cao, J. & Li, X. Image manipulation detection by multi-view multi-scale supervision. In *Proceedings of the IEEE/CVF international conference on computer vision*, 14185–14193 (2021).

[35] Shi, Z., Shen, X., Chen, H. & Lyu, Y. Global semantic consistency network for image manipulation detection. *IEEE Signal Process. Lett.* **27**, 1755–1759 (2020).

[36] Zhou, P. et al. Generate, segment, and refine: Towards generic manipulation segmentation. *In Proceedings of the AAAI conference on artificial intelligence* **34**, 13058–13065 (2020).

[37] Guo, X. *et al.* Hierarchical fine-grained image forgery detection and localization. In *Proceedings of the IEEE/CVF Conference on Computer Vision and Pattern Recognition*, 3155–3165 (2023).

[38] Wu, H., Zhou, J., Tian, J. & Liu, J. Robust image forgery detection over online social network shared images. In *Proceedings of the IEEE/CVF Conference on Computer Vision and Pattern Recognition*, 13440–13449 (2022).

[39] Ma, X., Du, B., Jiang, Z., Hammadi, A. Y. A. & Zhou, J. Iml-vit: Benchmarking image manipulation localization by vision transformer. arXiv preprint arXiv:2307.14863 (2023).

[40] Shao, Y., Wang, T. & Wang, L. Image manipulation detection based on irrelevant information suppression and critical information enhancement. *Eur. J. Artif. Intell.* 30504554241301395 (2025).

[41] Zhuo, L., Tan, S., Li, B. & Huang, J. Self-adversarial training incorporating forgery attention for image forgery localization. *IEEE Trans. Information Forensics Security* **17**, 819–834 (2022).

[42] Lou, Z., Cao, G., Guo, K., Weng, S. & Yu, L. Image forgery localization with state space models. arXiv preprint arXiv:2412.11214 (2024).

[43] Jiang, Y., Huang, Y., Chen, H. & Lyu, Y. Multi-modality boundary-guided network for generalizable image manipulation localization. *Multimed. Syst.* **31**, 1–13 (2025).

[44] Shao, Y., Dai, K. & Wang, L. Image tampering localization network based on multi-class attention and progressive subtraction. *Signal Image Video Process.* **19**, 2 (2025).

[45] Lou, Z., Cao, G., Guo, K., Yu, L. & Weng, S. Exploring multi-view pixel contrast for general and robust image forgery localization. *IEEE Trans. Informat. Forensics Security* (2025).

[46] Li, F., Pei, Z., Zhang, X. & Qin, C. Image manipulation localization using multi-scale feature fusion and adaptive edge supervision. *IEEE Trans. Multimed.* **25**, 7851–7866 (2022).

[47] Dou, L., Chen, M., Qiu, J. & Wang, J. Msf-net: Multi-stream fusion network for image manipulation detection and localization. *Digital Signal Process.* **161**, 105114 (2025).

[48] Dong, C., Chen, X., Hu, R., Cao, J. & Li, X. Mvss-net: Multi-view multi-scale supervised networks for image manipulation detection. *IEEE Trans. Pattern Anal. Machine Intell.* **45**, 3539–3553 (2022). 10.1109/TPAMI.2022.3180556 35671312

[49] Zhou, J., Ma, X., Du, X., Alhammadi, A. Y. & Feng, W. Pre-training-free image manipulation localization through non-mutually exclusive contrastive learning. In *Proceedings of the IEEE/CVF international conference on computer vision*, 22346–22356 (2023).

[50] Zhou, Y., Wang, H., Zeng, Q., Zhang, R. & Meng, S. A discriminative multi-channel noise feature representation model for image manipulation localization. In *ICASSP 2023-2023 IEEE International Conference on Acoustics, Speech and Signal Processing (ICASSP)*, 1–5 (IEEE, 2023).

[51] Wu, H., Zhou, J., Tian, J., Liu, J. & Qiao, Y. Robust image forgery detection against transmission over online social networks. *IEEE Trans. Inform. Forensics Security* **17**, 443–456 (2022).

[52] Zeng, Y., Zhao, B., Qiu, S., Dai, T. & Xia, S.-T. Toward effective image manipulation detection with proposal contrastive learning. *IEEE Trans. Circ. Syst. Video Technol.* **33**, 4703–4714 (2023).

[53] Liu, X., Liu, Y., Chen, J. & Liu, X. Pscc-net: Progressive spatio-channel correlation network for image manipulation detection and localization. *IEEE Trans. Circ. Syst. Video Technol.* **32**, 7505–7517 (2022).

[54] Kadam, K. D., Ahirrao, S. & Kotecha, K. [retracted] efficient approach towards detection and identification of copy move and image splicing forgeries using mask r-cnn with mobilenet v1. *Comput. Intell. Neurosci.* **2022**, 6845326 (2022). 35035463 10.1155/2022/6845326 PMC8754624

[55] Hu, X. *et al.* Span: Spatial pyramid attention network for image manipulation localization. In *Computer Vision–ECCV 2020: 16th European Conference, Glasgow, UK, August 23–28, 2020, Proceedings, Part XXI 16*, 312–328 (Springer, 2020).

[56] Su, L. *et al.* Can we get rid of handcrafted feature extractors? sparsevit: Nonsemantics-centered, parameter-efficient image manipulation localization through spare-coding transformer. arXiv preprint arXiv:2412.14598 (2024).

[57] Gao, Z. et al. Tbnet: A two-stream boundary-aware network for generic image manipulation localization. *IEEE Trans. Knowl. Data Eng.* **35**, 7541–7556 (2022).

[58] Li, S., Xu, S., Ma, W. & Zong, Q. Image manipulation localization using attentional cross-domain cnn features. *IEEE Trans. Neural Netw. Learn. Syst.* **34**, 5614–5628 (2021). 10.1109/TNNLS.2021.3130168 34855602

[59] Hao, J., Zhang, Z., Yang, S., Xie, D. & Pu, S. Transforensics: image forgery localization with dense self-attention. In *Proceedings of the IEEE/CVF International Conference on Computer Vision*, 15055–15064 (2021).

[60] Guillaro, F., Cozzolino, D., Sud, A., Dufour, N. & Verdoliva, L. Trufor: Leveraging all-round clues for trustworthy image forgery detection and localization. In *Proceedings of the IEEE/CVF conference on computer vision and pattern recognition*, 20606–20615 (2023).

[61] Guo, K., Cao, G., Lou, Z., Huang, X. & Liu, J. A lightweight and effective image tampering localization network with vision mamba. arXiv preprint arXiv:2502.09941 (2025).

[62] Singh, T., Goel, Y., Yadav, T. & Seniaray, S. Performance analysis of ela-cnn model for image forgery detection. In *2023 4th International Conference for Emerging Technology (INCET)*, 1–6 (IEEE, 2023).

[63] Tyagi, S. & Yadav, D. Forensicnet: Modern convolutional neural network-based image forgery detection network. *J. Forensic Sci.* **68**, 461–469 (2023). 36719038 10.1111/1556-4029.15210

[64] Song, H., Lin, B. & Ye, D. Tri-path backbone network for image manipulation localization. *IEEE Access* (2024).

[65] Xiao, B., Wei, Y., Bi, X., Li, W. & Ma, J. Image splicing forgery detection combining coarse to refined convolutional neural network and adaptive clustering. *Inform. Sci.* **511**, 172–191 (2020).

[66] Kwon, M.-J., Yu, I.-J., Nam, S.-H. & Lee, H.-K. Cat-net: Compression artifact tracing network for detection and localization of image splicing. In *Proceedings of the IEEE/CVF winter conference on applications of computer vision*, 375–384 (2021).

[67] Zhuang, P., Li, H., Tan, S., Li, B. & Huang, J. Image tampering localization using a dense fully convolutional network. *IEEE Trans. Inform. Forensics Security* **16**, 2986–2999 (2021).

[68] Cozzolino, D. & Verdoliva, L. Noiseprint: A cnn-based camera model fingerprint. *IEEE Trans. Inform. Forensics Security* **15**, 144–159 (2019).

[69] Bi, X., Zhang, Z. & Xiao, B. Reality transform adversarial generators for image splicing forgery detection and localization. In *proceedings of the IEEE/CVF international conference on computer vision*, 14294–14303 (2021).

